# A meta-analysis of public RNA-Seq data identifies conserved stress responses in rainbow trout

**DOI:** 10.1186/s12864-025-12127-2

**Published:** 2025-11-05

**Authors:** Sayed Mashequl Bari, Muntasim Fuad, Md Jubayer Hossain, Ishtiaque Ahammad, Mahmuda Begum, Md Mostofa Uddin Helal

**Affiliations:** 1 Department of Aquatic Animal Health Management, Sher-e-Bangla Agricultural University, Dhaka, 1207 Bangladesh; 2 Big Bioinformatics Lab, Center for Health Innovation, Research, Action, and Learning, Dhaka, 1207 Bangladesh; 3 Bioinformatics Division National Institute of Biotechnology, Dhaka, 1349 Bangladesh; 4 Biological Research Division, Bangladesh Council of Scientific and Industrial Research, Dhaka, 1205 Bangladesh; 5 College of Agriculture, Shanxi Agricultural University, Linfen, China

**Keywords:** Rainbow trout, Heat stress, Hypoxia, Meta-analysis, Transcriptome, DEGs

## Abstract

**Background:**

Rainbow trout (*Oncorhynchus mykiss*), a commercial aquaculture species, experiences physiological stress under environmental challenges. While several bulk RNA-Seq studies have investigated heat and hypoxic stress responses in rainbow trout, a comprehensive analysis identifying common genes and pathways is still lacking. This study addresses this need by analyzing four public RNA-Seq datasets from *O. mykiss* to identify conserved molecular responses to these stress.

**Result:**

The meta-analysis identified 1,006 (555 upregulated and 451 downregulated) protein-coding differentially expressed genes (DEGs). Among them, five genes, *cdkn1ba*, *znf395a*, *klf9*, *irs2b*, and *tsc22d3*, were commonly regulated under both stress conditions, indicating their shared roles in cellular homeostasis. The meta-analysis revealed highly regulated heat shock protein (hsp) family genes and hypoxia-inducible factor (HIF) genes. Gene ontology (GO) enrichment analysis revealed that DEGs under heat and hypoxia stress were associated primarily with the cell cycle, DNA metabolic processes, catalytic activity, and membrane components. Pathway enrichment of these genes revealed coordinated activation of protein processing in the endoplasmic reticulum, p53-mediated cell-cycle arrest, FoxO signaling and oxidative stress responses, indicating a shared core program for proteostasis and damage control.

**Conclusion:**

This meta-analysis revealed conserved gene expression patterns and key regulatory pathways driving rainbow trout adaptation to heat and hypoxia, providing valuable insights for enhancing stress resistance in aquaculture.

**Supplementary Information:**

The online version contains supplementary material available at 10.1186/s12864-025-12127-2.

## Background

The rainbow trout (*O. mykiss*) is a cold-water aquaculture species with significant economic and ecological importance [1]. However, they are susceptible to abiotic stressors like heat [2] and hypoxia [3], which can negatively impact their physiology, immune function, and overall productivity. This species is widely farmed in freshwater environments across Europe and North America, thriving within a temperature range of 12–18 °C and requiring a dissolved oxygen level of approximately 8 mg/L for optimal health. However, when exposed to temperatures near 25 °C and dissolved oxygen levels below 4.5–3 mg/L, rainbow trout become vulnerable [4]. In recent decades, global warming and thermal shifts have presented significant challenges to commercial fish culture [5]. The increasing frequency of extreme heatwaves disrupts dissolved oxygen concentrations [6] and adversely affects fish growth, reproduction, and survival, heightening their susceptibility to both biotic and abiotic stressors [7, 8]. Rainbow trout are particularly at risk from climate change, seasonal fluctuations, and rising water temperatures in various aquaculture systems [9].

To cope with abiotic stress and maintain normal physiology, salmonid fishes like rainbow trout activate adaptive mechanisms primarily through gene expression [10]. In response to heat stress, they upregulate heat shock genes that encode various heat shock proteins (HSPs). For example, rainbow trout express *hsp70* and *hsp90* [11], while redband trout (*Oncorhynchus mykiss gairdneri*) express *hsp47* alongside *hsp70* and *hsp90* [12] in response to thermal stress. Similarly, Atlantic salmon (*Salmo salar*) and Chinook salmon (*Oncorhynchus tshawytscha*) show upregulation of the *hsp90a* and *serpinh1* genes [13, 14]. These HSPs basically act as molecular chaperones that ensure proper protein folding, prevent the aggregation of misfolded proteins, and assist in repairing or degrading damaged proteins [15]. Interestingly, these stress-induced proteins function either in independent or collaborative ways within various intracellular signaling pathways, including the protein and endoplasmic reticulum stress response [16], general metabolic processes [17], and cellular functions such as molecular binding and membrane integrity [18] in rainbow trout. In addition, fish survival under hypoxia depends on metabolic rate reduction and the activation of hypoxia-inducible genes, primarily regulated by hypoxia-inducible factors (HIFs) [19, 20]. In rainbow trout, adaptation to low-oxygen environments involves modulation of genes associated with changes in liver antioxidant metabolism, suppression of the cell cycle [21], inflammatory responses [10], and metabolic reprogramming [22].

The rainbow trout, often referred to as an aquatic lab rat, is a valuable model for various biological studies investigating stress responses [23]. Next-generation RNA-Seq serves as a powerful tool for examining stress-responsive DEGs , their encoded proteins, and the biological pathways involved in responses to thermal stress and anoxic conditions [24]. While several transcriptomic studies have explored the responses of rainbow trout to heat and hypoxia using high-quality Illumina sequencing, vary significantly in focus of experimental design, and data processing pipelines, which limit the reliability of cross-study comparisons. For example, Zhou et al. [25] identified lncRNA–mRNA regulatory pairs related to immune regulation, apoptosis, and metabolism under acute heat stress but limited their analysis to head kidney tissue and a single exposure duration. In another study, Sun et al. [26] reported numerous alternative splicing events in liver tissue under heat stress but did not evaluate their persistence across different conditions or individuals. Similarly, Wu et al. [27] examined metabolic and signaling changes during hypoxia and reoxygenation in the liver, while another study by Wu et al. [28] focused on lncRNA–miRNA–mRNA networks in hypoxia, emphasizing anaerobic metabolism and immune suppression; however, neither study incorporated heat stress responses.

Although these studies offer valuable insights, they often present divergent sets of DEGs with minimal overlap across conditions. To address these gaps, we conducted a systematic meta-analysis [29] of four publicly available RNA-Seq datasets involving heat and hypoxia exposure in rainbow trout. Our goal was to identify conserved DEGs and shared biological pathways that highlights general stress adaptation in this species, thereby providing new insights into the mechanisms of stress adaptation.

## Methods

### Gene expression data acquisition

Gene expression data related to heat stress and hypoxia were retrieved from the NCBI Gene Expression Omnibus (GEO) database (https://www.ncbi.nlm.nih.gov/geo/) [30]. To refine the search, we used keywords such as “rainbow trout,” “*Oncorhynchus mykiss*,” “heat stress,” “heat shock,” “thermal stress,” “heat-shock protein,” “hypoxia,” “hypoxic condition,” “anoxic condition,” and “anoxia,” along with the study type “expression profiling by high-throughput sequencing.” To ensure high quality and minimize technical bias, we selected RNA-Seq datasets from public repositories that were generated on the Illumina platform using fish weighing 200–400 g, collected under controlled conditions with at least three replicates, and were supported by metadata and detailed methodologies.

This meta-analysis incorporated four high-quality datasets: two heat stress and two hypoxic stress datasets. Each dataset was comprised of a minimum of three control samples and three to nine treatment samples. The four datasets (PRJNA1092638, PRJNA559610, PRJNA1064938, and PRJNA1000995) comprised 30 paired-end SRA samples, including 12 controls and 18 stress treatments. The anoxic samples were divided into chronic (4.5 mg/L oxygen) and acute (3 mg/L oxygen) groups, with controls at 8 mg/L. Heat stress samples were collected at 18 °C (control) and 24 °C (stress) after seven days. Detailed information on the RNA-Seq datasets, including the GEO ID, BioProject, SRA run, stress type, temperature, tissue, sampling duration, and the reference genome, is provided in Additional File **1**: Table **S1**.

### Data pre-processing

Raw fastq files from the Sequence Read Archive (SRA) were downloaded from NCBI GEO (https://www.ncbi.nlm.nih.gov/gds/) via SRAtools v3.2.1. Quality checks were performed with FastQC v0.12.1 [31], followed by trimming of low-quality bases and adapters via Trim Galore v0.6.3 (https://github.com/FelixKrueger/TrimGalore) and Cutadapt [32]. Cleaned reads were rechecked with FastQC and then aligned to the rainbow trout reference genome (Omyk_1.1) from Salmobase (https://salmobase.org/genomes/RainbowTrout/USDA_OmykA_1.1/overview) via HISAT2 v2.2.1 [33]. Sequence alignment files (SAM) were converted to sorted binary alignment files (BAM) with SAMtools v1.21 [34]. Transcript abundance was estimated with Stringtie v3.0.0 [35, 36], and FPKM (Fragments Per Kilobase of transcript per Million mapped reads) value was used to quantify transcript-level gene expression [37]. For differential expression analysis, read counts were obtained from sorted BAM files with Subread v2.0.8 (http://subread.sourceforge.net/), which were aligned against the OmykA_1.1 genomic annotation.

### Differential gene expression analysis

Differential expression analysis was performed via the DESeq2 package (v1.30.1) in R 4.0.5 [38] (https://bioconductor.org/packages/release/bioc/html/DESeq2.html), and the negative binomial model was applied to normalize the raw read counts. Low-count genes were removed via DESeq2 default independent filtering. DEGs with adjusted P values < 0.05 (Benjamini‒Hochberg correction) and |log_2_FC| >1 were identified in each dataset. The surrogate variable analysis (SVA) using the R package sva (v3.50.0) were used to reduce potential batch effects. Principal Component Analysis (PCA) was performed on the expression matrices before and after batch effect removal to verify the effectiveness of batch effect corrections (Supplementary Figure S1). Shared and unique DEGs across the heat stress and hypoxia datasets were visualized via the VennDiagram package (v1.7.3) [39] (https://github.com/uclahs-cds/package-VennDiagram). To visualize the transcript level expression patterns, heatmaps were generated with ComplexHeatmap (v2.15.4) [40] (https://bioconductor.org/packages/release/bioc/html/ComplexHeatmap.html) via log_2_(FPKM + 1) transformed values. This transformation stabilized the variance and reduced the impact of zero-count values. The expression matrices were subsets to include only DEGs identified under each condition (adjusted P value < 0.05, |log_2_FC| >1). Hierarchical clustering was performed via Euclidean distance and complete linkage, with samples annotated by treatment.

### Meta-analysis

A meta-analysis was performed via MetaVolcanoR (v1.4.0) (https://github.com/csbl-usp/MetaVolcanoR) to integrate DEG results across the four datasets. The random effect model (REM) was applied to combine log_2_(fold changes) while accounting for variance, generating summary p values. For the heat and hypoxia datasets, Fisher’s method was used to combine adjusted p values. The top 1% of the most consistently perturbed genes were identified via the ‘metathr’ parameter. Ensembl gene IDs for *O. mykiss* were annotated with the biomaRt (v3.21) (https://bioconductor.org/packages/release/bioc/html/biomaRt.html) R package. DEGs were defined as those with adjusted *P* < 0.05 and |log_2_FC| >1. Genes with log_2_FC >1 were considered significantly upregulated, whereas those with log_2_FC < − 1 were classified as significantly downregulated. Volcano plots were generated via ggplot2 (v3.5.2) (https://ggplot2.tidyverse.org/) [41] to visualize significant genes.

### Gene ontology (GO) enrichment analysis

The GO enrichment analysis of DEGs was performed using the GO database (http://www.geneontology.org) [42] via the g:Profilerv(0.2.3) R package (https://cran.r-project.org/web/packages/gProfileR/index.html) [43]. GO terms with an adjusted p value < 0.05, corrected by the Benjamini–Hochberg false discovery rate (FDR) [44], were considered statistically significant.

### KEGG pathway enrichment analysis

The Kyoto Encyclopedia of Genes and Genomes (KEGG) database (http://www.genome.jp/kegg/pathway.html) [45] was used for the DEG pathway analysis. The clusterProfiler (https://bioconductor.org/packages/release/bioc/html/clusterProfiler.html) R package was used to test the statistical enrichment of DEGs in KEGG pathways. Pathways with an adjusted p value < 0.05 (Benjamini‒Hochberg (BH) corrected) were used as a threshold for significantly enriched pathways.

## Results

### Retrieved RNA-Seq datasets

**Fig. 1:**
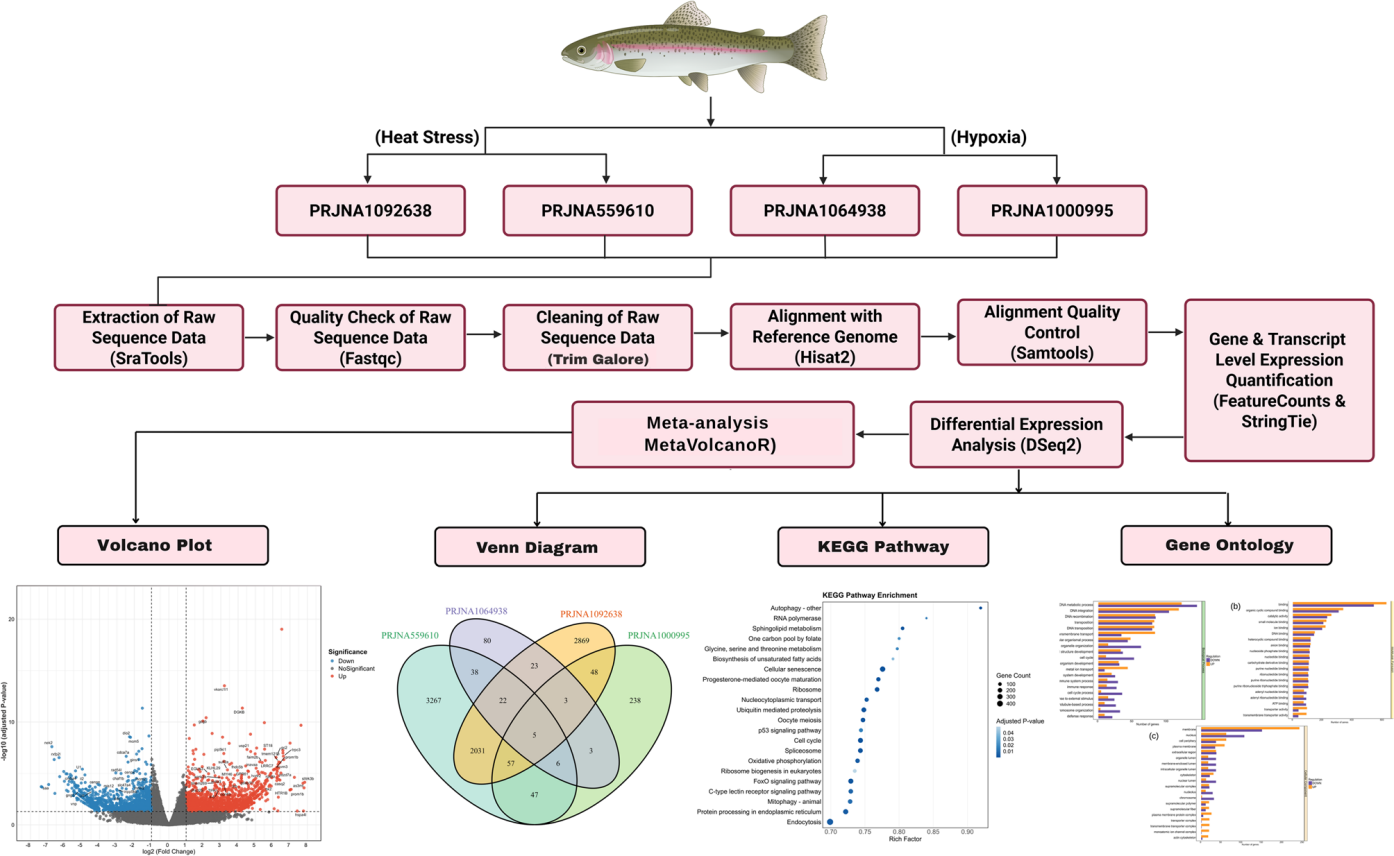
Meta-analysis workflow to investigate high-quality RNA-Seq datasets of rainbow trout

To investigate the transcriptomic responses of *O. mykiss*, four publicly available RNA-Seq datasets derived from liver and muscle tissues exposed to heat and hypoxic stress were curated and analyzed (Table 1). These datasets were retrieved from the NCBI Sequence Read Archive (SRA) and subjected to a standardized meta-analysis pipeline (Fig. 1). The selected datasets included two heat stress experiments (PRJNA1092638 and PRJNA559610) and two hypoxia related experiments (PRJNA1064938 and PRJNA1000995), each comprising six to nine samples. The rainbow trout reference genome OmykA_1.1_genomic.fna and gene set OmykA_1.1_genomic.gff, including 64,494 coding and noncoding genes, were used for expression quantification. The detailed information of RNA-Seq datasets, including GEO ID, Bio Project, SRA run, stress type, temperature, tissue, sampling duration, and reference genome, are provided in supplementary table **S**1.

**Table 1:** RNA-Seq meta-analysis datasets in response to heat and hypoxia stress in rainbow trout

Bio Project	Accession Number		Biological Characteristics				Technical Characteristics		References
	Batch ID	Sample ID	Tissue	Weight (gm)	Condition	Time	Platform	Reference Genome	
PRJNA1092638	GSE262612	GSM8172384	Liver	400	18 °C	7 days	Illumina NovaSeq 6000	Omyk_1.1	[46], [47]
		GSM8172385	Liver	400	18 °C	7 days			
		GSM8172386	Liver	400	18 °C	7 days			
		GSM8172387	Liver	400	24 °C	7 days			
		GSM8172388	Liver	400	24 °C	7 days			
		GSM8172389	Liver	400	24 °C	7 days			
PRJNA559610	GSE135668	GSM4025692	Liver	200	18 °C	7 days	Illumina HiSeq 4000		[27]
		GSM4025693	Liver	200	18 °C	7 days			
		GSM4025694	Liver	200	18 °C	7 days			
		GSM4025695	Liver	200	24 °C	7 days			
		GSM4025696	Liver	200	24 °C	7 days			
		GSM4025697	Liver	200	24 °C	7 days			
PRJNA1064938	GSE253274	GSM8017523	Muscle	200	4.5 mg/L	12 h	Illumina NovaSeq 6000		NA
		GSM8017524	Muscle	200	4.5 mg/L	12 h			
		GSM8017525	Muscle	200	4.5 mg/L	12 h			
		GSM8017526	Muscle	200	3 mg/L	12 h			
		GSM8017527	Muscle	200	3 mg/L	12 h			
		GSM8017528	Muscle	200	3 mg/L	12 h			
		GSM8017529	Muscle	200	8.5 mg/L	12 h			
		GSM8017530	Muscle	200	8.5 mg/L	12 h			
		GSM8017531	Muscle	200	8.5 mg/L	12 h			
PRJNA1000995	GSE239806	GSM7673627	Liver	200	4.5 mg/L	12 h	Illumina HiSeq 4000		[48]
		GSM7673628	Liver	200	4.5 mg/L	12 h			
		GSM7673629	Liver	200	4.5 mg/L	12 h			
		GSM7673630	Liver	200	3 mg/L	12 h			
		GSM7673631	Liver	200	3 mg/L	12 h			
		GSM7673632	Liver	200	3 mg/L	12 h			
		GSM7673633	Liver	200	8.5 mg/L	12 h			
		GSM7673634	Liver	200	8.5 mg/L	12 h			
		GSM7673635	Liver	200	8.5 mg/L	12 h

### Expression profiling under stress conditions

**Fig. 2:**
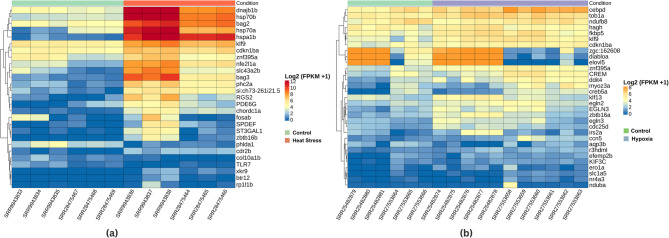
Heatmap showing log₂(FPKM + 1) expression levels across samples. **A** Samples are color coded as green (control) or orange (heat stress) **B** samples are color coded as green (control) or purple (hypoxia). Genes were hierarchically clustered on the basis of expression similarity, revealing distinct transcriptional profiles between the control and stress groups

To examine transcript-level responses to heat and hypoxic stress, two heatmaps (Fig. 2) were generated using log2(FPKM + 1) values across 30 samples. FPKM (Fragments Per Kilobase of transcript per Million mapped reads) is a normalization method in RNA-seq that accounts for gene length and sequencing depth, enabling accurate comparison of expression levels across genes and samples. Hierarchical clustering was performed on both genes and samples, as shown by the dendrograms above each heatmap, to assess whether samples group by treatment as expected. Under heat stress (Fig. 2a), a distinct cluster of genes, including *dnajb1b*, *hsp70a/b*, *hspa1b*, and *bag2/3*, exhibited strong upregulation compared to controls, indicating a classical heat shock response. Notably, the dendrogram shows clear and robust clustering of samples by treatment group: all heat-stressed samples (orange) form a cohesive cluster that is distinctly separated from the control samples (green). This demonstrates that the heat stress treatment is the primary driver of gene expression variation in these samples.

In response to hypoxia (Fig. 2b), genes such as *cebpd*, *egln2/3*, *klf9*, and *cdkn1ba* were notably upregulated, with hypoxia-treated samples (purple) and control samples (green) forming predominantly distinct clusters in the dendrogram. However, clustering by treatment in the hypoxia group shows slightly more heterogeneity than in the heat stress group; one or two samples do not strictly segregate according to treatment. This minor overlap may be due to inherent biological variability among individuals, variation in treatment response, or technical factors such as differences in RNA quality or sequencing depth. Such imperfect clustering is not uncommon in transcriptomics studies and does not affect the observation that treatment is the major source of expression variation. A complete list of FPKM values for all expressed genes across the samples is provided in Supplementary Table S2.

### Venn diagram of cumulative differential gene expression

**Fig. 3:**
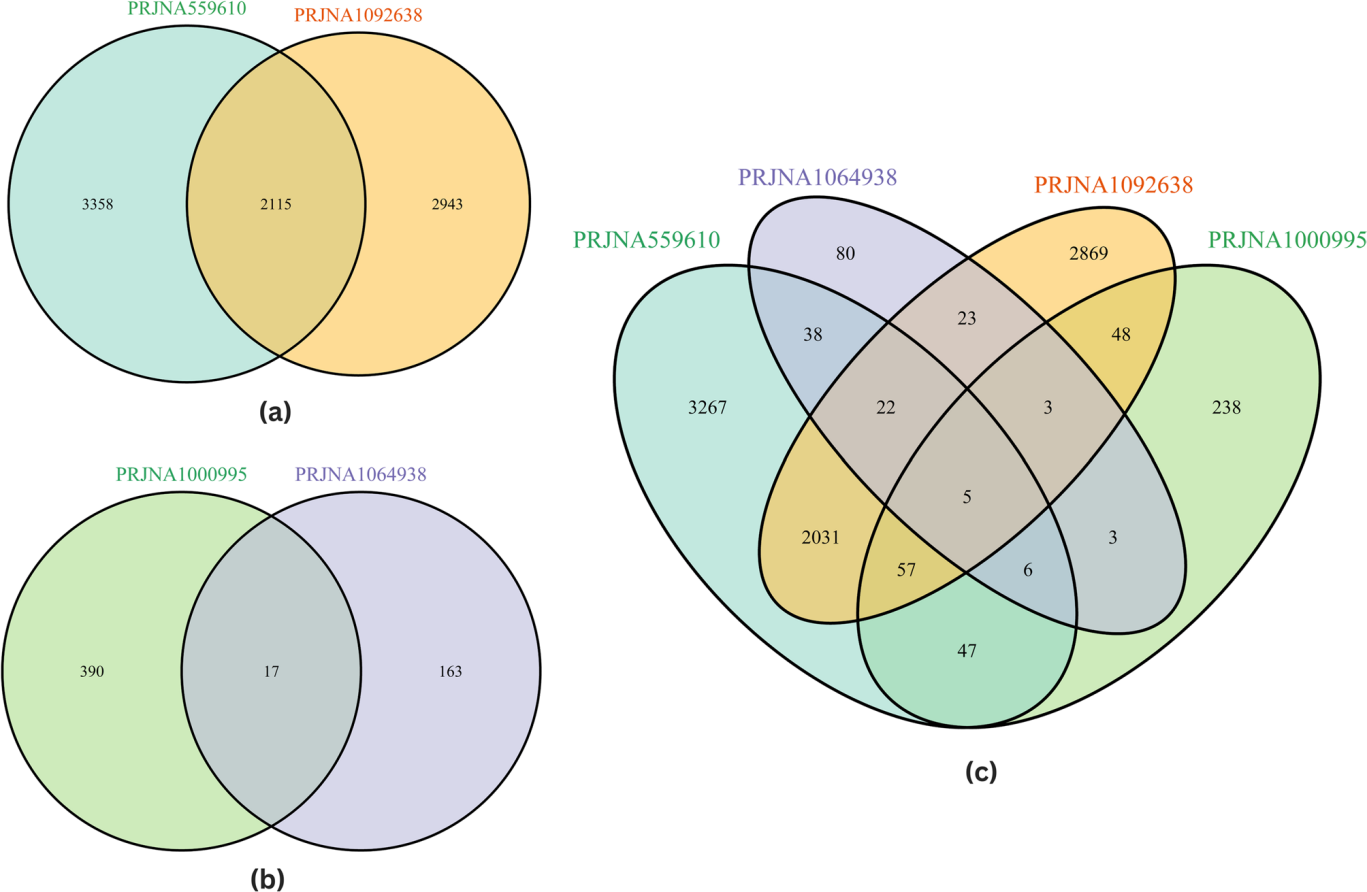
Venn diagrams illustrating the shared DEGs under thermal and hypoxic stress conditions. **A** Overlap of DEGs between two thermal stress RNA-Seq datasets (PRJNA559610 in greenish-blue and PRJNA1092638 in orange) **B** overlap of DEGs between two hypoxic stress RNA-Seq datasets (PRJNA1000995 in light green and PRJNA1064938 in purple), and **C** comprehensive four-set venn diagram combining both thermal (PRJNA559610 and PRJNA1092638) and hypoxic (PRJNA1000995 and PRJNA1064938) stress datasets. All genes included had an adjusted p value < 0.05 and an absolute log₂-fold change > 1

Venn diagram analysis (Fig. 3) revealed both unique and overlapping patterns of DEGs across the heat and hypoxia stress datasets. A comparison of the two heat stress datasets (PRJNA559610 and PRJNA1092638) revealed that 2,115 DEGs were shared, whereas 3,358 and 2,943 DEGs were uniquely expressed in PRJNA559610 and PRJNA1092638, respectively (Fig. 3a), suggesting both conserved and dataset-specific responses to heat stress. For the hypoxia datasets (PRJNA1000995 and PRJNA1064938), only 17 overlapping DEGs were identified, with 390 and 163 DEGs unique to each dataset, respectively (Fig. 3b), indicating a more divergent transcriptional response under hypoxia. The integrated venn diagram (Table 2; Fig. 3c) revealed five DEGs (*cdkn1ba*, *znf395a*, *klf9*, *irs2b*, and *tsc22d3*) that were commonly found across all four stress conditions. A detailed list of combined intersecting genes is provided in supplementary table **S3**.

**Table 2:** Commonly expressed genes among the four RNA-seq datasets

SN	Ensemble ID	Gene Symbol	Annotation	Adj *P*	Average_ Log_2_FC	Effect
1	ENSOMYG00000017016	*cdkn1ba*	Cyclin dependent Kinase inhibitor 1ba	1.94568E-10	1.469990741	Up
2	ENSOMYG00000022552	*znf395a*	Zinc finger protein 395	6.04783E-08	1.397240041	Up
3	ENSOMYG00000028743	*klf9*	Kruppel like factor 9	8.05094E-11	1.965697087	Up
4	ENSOMYG00000045565	*irs2b*	insulin receptor substrate 2b	5.66E-11	1.479284258	Up
5	ENSOMYG00000059646	*tsc22d3*	TSC22 domain family member 3	1.68E-200	0.643931804	Up

### Meta-analysis of cumulative transcriptome data

Meta-analysis of the top 40 DEGs (20 upregulated and 20 downregulated; Table 3) revealed shared molecular responses to heat and hypoxia stress in rainbow trout. From the complete dataset, a total of 1,006 annotated DEGs were identified, including 555 upregulated and 451 downregulated genes. Among the top 20 upregulated genes were *hspa1b*, *hspa4l*, and *hk2*, reflecting enhanced proteostasis and glycolytic reprogramming. Additionally, the upregulation of *scn1ba*, *trpc3*, and *casq2* suggested increased ion transport and calcium handling, potentially supporting cellular excitability and survival under thermal and hypoxic challenges. In contrast, several genes, including *saa*, *nek2*, *lin28b*, and *pgm3*, were significantly downregulated, implying the suppression of immune responses, cell cycle progression, and metabolic activity. The complete list of DEGs can be found in supplementary table **S**4.

**Table 3:** List of the top DEGs identified in the meta-analysis. Each row represents a gene with its unique ensemble gene ID, gene symbol, log_2_-fold change (log_2_FC), adjusted p value (adj. P), direction of regulation (up or down), and a brief gene description

SN	Gene ID	Gene Symbol	log2Fold Change	adj.*P*	Regula tion	Gene Description
1.	ENSOMYG00000029610	*scn1ba*	20.72375903	1.0585E-25	UP	sodium channel, voltage-gated, type I, beta a
2.	ENSOMYG00000010447	*hspa1b*	8.134592588	0.041275173	UP	heat shock protein family A (Hsp70) member 1B
3.	ENSOMYG00000005025	*slitrk3b*	7.849197611	7.18516E-05	UP	SLIT and NTRK-like family, member 3b
4.	ENSOMYG00000034757	*hspa4l*	7.41921313	0.044692546	UP	heat shock protein 4 like
5.	ENSOMYG00000029994	*as3mt*	7.063315689	0.000354443	UP	arsenite methyltransferase
6.	ENSOMYG00000054931	*prom1b*	7.009340574	0.000393343	UP	prominin 1 b
7.	ENSOMYG00000023837	*HTR1B*	7.000090794	0.000400397	UP	5-hydroxytryptamine receptor 1B
8.	ENSOMYG00000036455	*trpc3*	6.61811721	2.103E-07	UP	transient receptor potential cation channel, subfamily C, member 3
9.	ENSOMYG00000018660	*casq2*	6.416615099	4.40883E-05	UP	calsequestrin 2
10.	ENSOMYG00000062678	*tmem121b*	6.352664125	7.66057E-07	UP	transmembrane protein 121B
11.	ENSOMYG00000021686	*pdzd7a*	6.257184426	6.36944E-06	UP	PDZ domain containing 7a
12.	ENSOMYG00000044394	*rcvrn3*	6.249410964	4.76288E-06	UP	recoverin 3
13.	ENSOMYG00000001466	*gc2*	6.193889296	2.77611E-07	UP	guanylyl cyclase 2
14.	ENSOMYG00000053172	*prom1b*	6.135962395	3.72423E-06	UP	prominin 1 b
15.	ENSOMYG00000032225	*hk2*	5.832901366	0.000132129	UP	hexokinase 2
16.	ENSOMYG00000015551	*CA12*	5.637990086	0.001021395	UP	carbonic anhydrase 12
17.	ENSOMYG00000015268	*SORCS1*	5.591489714	0.001138484	UP	sortilin related VPS10 domain containing receptor 1
18.	ENSOMYG00000068944	*rbp1.1*	5.583588416	5.1952E-05	UP	retinol binding protein 1, cellular, tandem duplicate 1
19.	ENSOMYG00000043818	*PURG*	5.564399942	0.000107922	UP	purine rich element binding protein G
20.	ENSOMYG00000025223	*ST18*	5.531857722	4.34161E-08	UP	ST18 C2H2C-type zinc finger transcription factor
21.	ENSOMYG00000019786	*alx1*	−4.602910798	0.014122604	DOWN	ALX homeobox 1
22.	ENSOMYG00000054259	*mettl25*	−4.630552329	0.002410291	DOWN	methyltransferase like 25
23.	ENSOMYG00000020940	*pgm3*	−4.642976858	0.005804978	DOWN	phosphoglucomutase 3
24.	ENSOMYG00000000542	*LIN28B*	−4.658682499	0.006284585	DOWN	lin-28 homolog B
25.	ENSOMYG00000037656	*zgc:162,592*	−4.758909738	0.000945816	DOWN	zgc:162,592
26.	ENSOMYG00000005318	*hs3st3l*	−4.763724981	0.004096918	DOWN	heparan sulfate (glucosamine) 3-O-sulfotransferase 3-like
27.	ENSOMYG00000043108	*slc45a1*	−4.781419783	0.000318668	DOWN	solute carrier family 45 member 1
28.	ENSOMYG00000031339	*atp2b3a*	−4.910512158	0.007065779	DOWN	ATPase plasma membrane Ca2 + transporting 3a
29.	ENSOMYG00000053698	*gng3*	−5.173125724	0.00049838	DOWN	guanine nucleotide binding protein (G protein), gamma 3
30.	ENSOMYG00000064868	*MINAR1*	−5.31377575	0.000166115	DOWN	membrane integral NOTCH2 associated receptor 1
31.	ENSOMYG00000061140	*tmtops3a*	−5.326037077	0.002432341	DOWN	teleost multiple tissue opsin 3a
32.	ENSOMYG00000010229	*hsd17b2*	−5.355942618	0.000142353	DOWN	hydroxysteroid (17-beta) dehydrogenase 2
33.	ENSOMYG00000067015	*nphs1*	−5.363997998	0.000160952	DOWN	NPHS1 adhesion molecule, nephrin
34.	ENSOMYG00000018854	*ebpl*	−5.444751747	0.000109371	DOWN	EBP like
35.	ENSOMYG00000003145	*cryba4*	−5.484970208	7.34676E-05	DOWN	crystallin, beta A4
36.	ENSOMYG00000018645	*aqp8b*	−5.50415504	0.000548403	DOWN	aquaporin 8b
37.	ENSOMYG00000024267	*adorb1a*	−5.596001261	0.000272998	DOWN	adenosine receptor B1a
38.	ENSOMYG00000014689	*rxfp2l*	−6.344529487	2.8946E-07	DOWN	relaxin family peptide receptor 2, like
39.	ENSOMYG00000000907	*nek2*	−6.760271277	2.50056E-08	DOWN	NIMA-related kinase 2
40.	ENSOMYG00000061342	*saa*	−6.947294466	0.000118632	DOWN	serum amyloid A

**Fig. 4:**
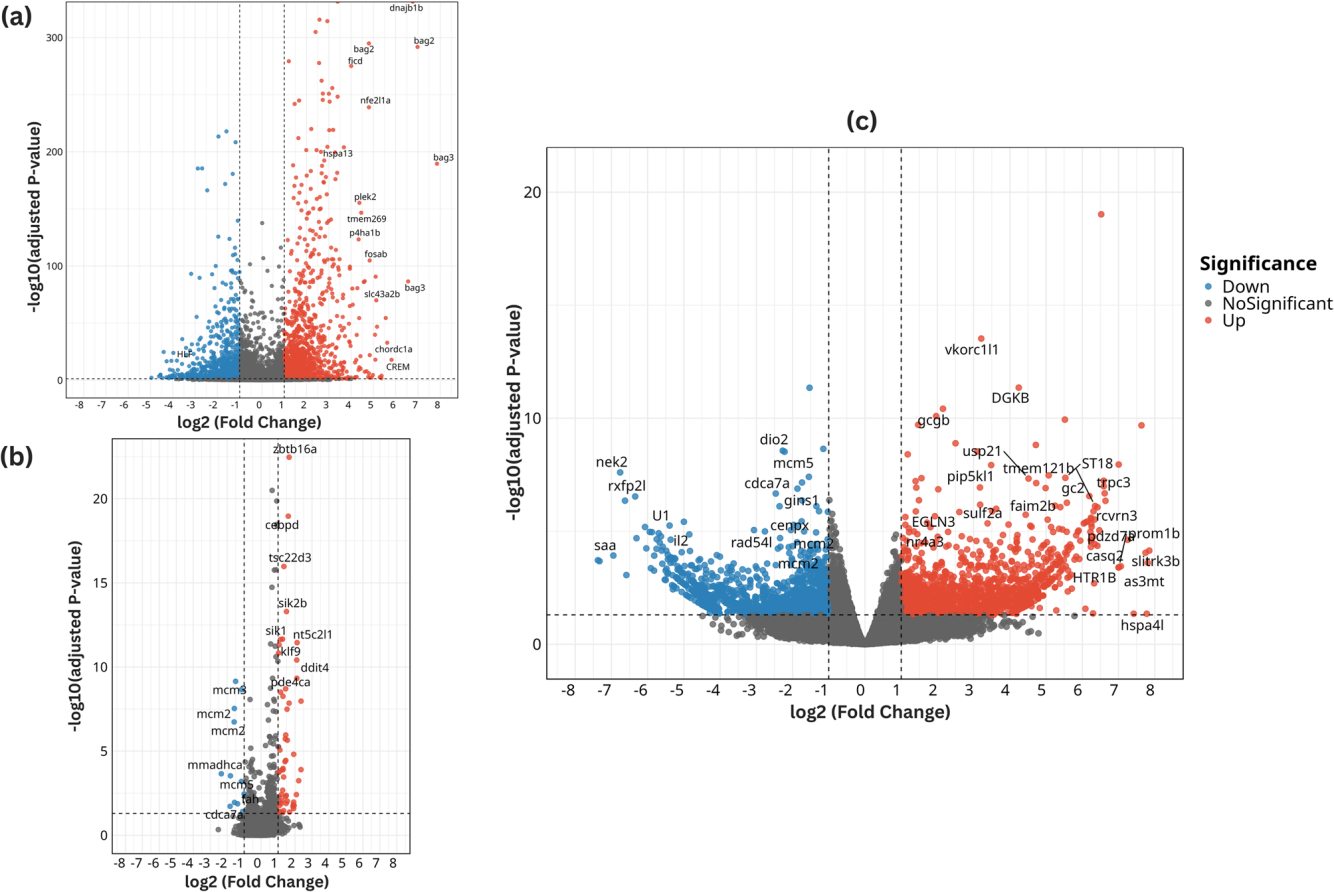
Volcano plots illustrating DEGs in rainbow trout (*O. mykiss*) under heat and hypoxic stress. Each plot presents the log₂ fold change (x-axis) against the –log₁₀ adjusted p-value (y-axis) **A** combined analysis of two heat stress datasets **B** combined analysis of two hypoxia stress datasets; and **C** cumulative analysis of all four datasets. Significantly upregulated genes (log₂FC > 1, adjusted *p* < 0.05) are shown in red, downregulated genes (log₂FC < − 1, adjusted *p* < 0.05) are shown in blue, and non-significant genes are shown in gray

The volcano plot analysis in Fig. 4 offers a visual snapshot of differential gene expression, summarizing both the magnitude and significance of gene regulation across conditions. In the heat stress analysis (Fig. 4a), genes such as *dnajb1b*, *hspa13*, *fosab*, *bag2*, and *chordc1a* were upregulated, underscoring their key roles in protein folding and cellular stress management. Under hypoxic stress (Fig. 4b), highly expressed upregulated genes included *ddit4*, *tsc22d3*, *klf9*, and *cebpd*, reflecting crucial adaptive mechanisms to low oxygen availability. The cumulative meta-analysis volcano plot (Fig. 4c), which integrates data from multiple tissues and both stress conditions, revealed a broader and more consistent set of regulated genes, highlighting *hspa4l*, *trpc3*, *casq2*, *prom1b*, and *htr1b* as among the most significantly upregulated. This pattern reinforces the importance of heat shock proteins, calcium signaling, and neural processes in rainbow trout’s stress resilience. Whereas, several genes, including *saa* (inflammation suppression), *nek2*, *mcm5* (cell cycle inhibition), and *dio2* (thyroid hormone regulation), were strongly downregulated.

### Gene ontology (GO) analysis

**Fig. 5:**
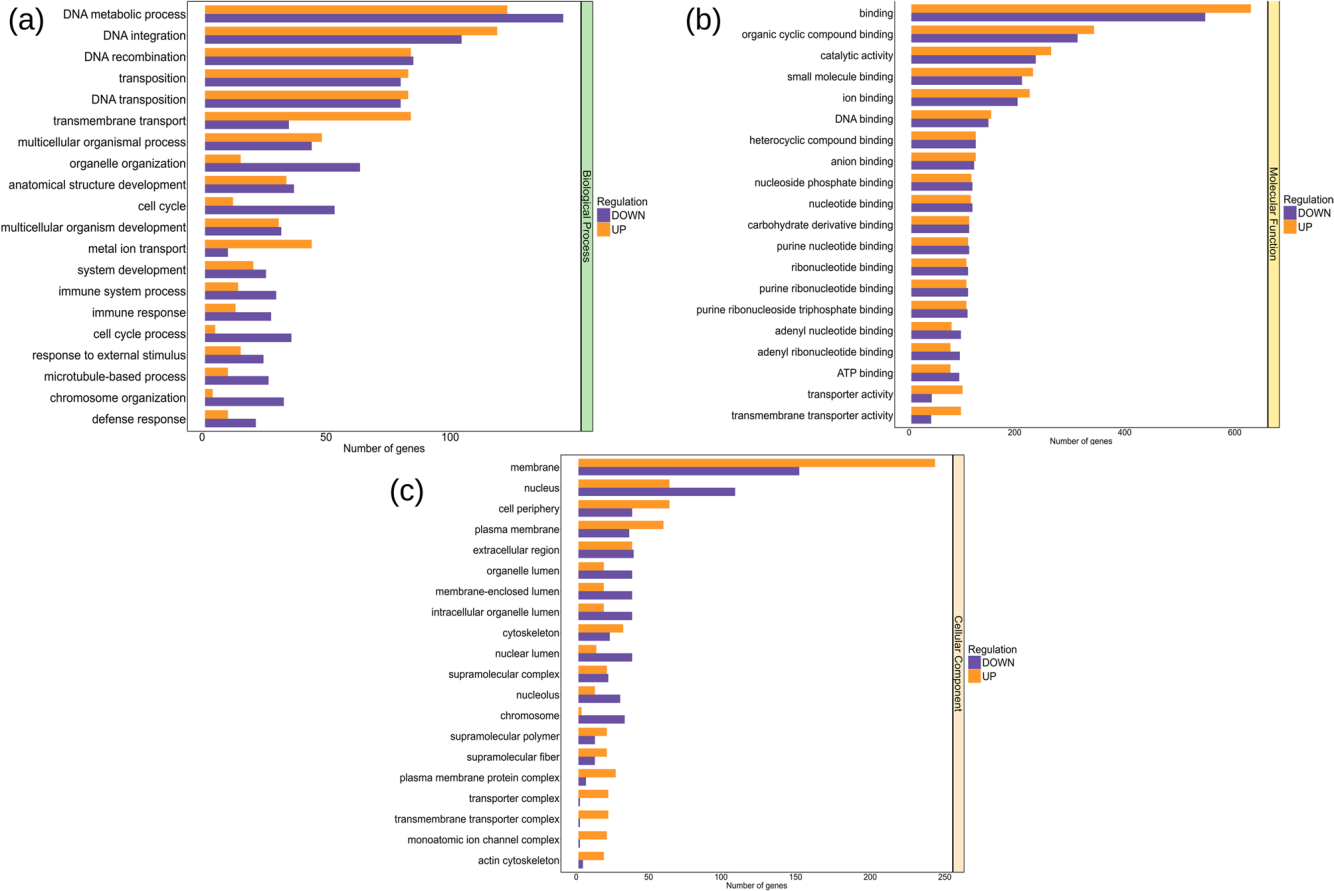
Gene Ontology (GO) enrichment analysis of DEGs under heat and hypoxia stress in rainbow trout. The analysis results were categorized into **A** biological processes (BP), **B** molecular functions (MF), and **C** cellular components (CC) categories. Bars represent the number of significantly upregulated (orange), and downregulated (purple) genes associated with each GO term. Only the top enriched GO terms with adjusted p-values < 0.05 are displayed for each category

Gene Ontology (GO) enrichment analysis of the DEGs revealed distinct patterns of biological adaptation under heat and hypoxia stress in rainbow trout liver and muscle tissues (Fig. 5; Table 4). The combined DEGs included 123 “biological processes,” 48 “cell components,” and 79 “molecular functions” terms. A complete list of 250 terms is provided in supplementary table **S5**.

**Table 4:** List of the top 30 gene ontology (GO) terms enriched in the meta-analysis of the four RNA-seq datasets

SN	GO ID	GO Term Description	GO Source	Genes of GO Term Present in Data	Adjust *p-*value
1.	GO:0006259	DNA metabolic process	BP	260	5.497552e-04
2.	GO:0015074	DNA integration	BP	216	4.194433e-02
3.	GO:0006310	DNA recombination	BP	163	7.567125e-03
4.	GO:0032196	transposition	BP	157	1.334342e-02
5.	GO:0006313	DNA transposition	BP	157	1.334342e-02
6.	GO:0055085	transmembrane transport	BP	114	8.257490e-03
7.	GO:0032501	multicellular organismal process	BP	88	6.289251e-03
8.	GO:0006996	organelle organization	BP	75	3.105766e-03
9.	GO:0048856	anatomical structure development	BP	67	4.743953e-02
10.	GO:0007049	cell cycle	BP	62	7.558401e-13
11.	GO:0005488	binding	MF	1151	2.556815e-13
12.	GO:0097159	organic cyclic compound binding	MF	634	2.905729e-07
13.	GO:0003824	catalytic activity	MF	480	4.562375e-03
14.	GO:0036094	small molecule binding	MF	422	1.953388e-05
15.	GO:0043167	ion binding	MF	408	3.171005e-05
16.	GO:0003677	DNA binding	MF	285	2.905729e-07
17.	GO:1,901,363	heterocyclic compound binding	MF	234	3.930839e-06
18.	GO:0043168	anion binding	MF	231	1.237496e-06
19.	GO:1,901,265	nucleoside phosphate binding	MF	220	1.953388e-05
20.	GO:0000166	nucleotide binding	MF	219	1.958613e-05
21.	GO:0016020	membrane	CC	387	8.785775e-17
22.	GO:0005634	nucleus	CC	166	2.907332e-02
23.	GO:0071944	cell periphery	CC	97	3.601206e-06
24.	GO:0005886	plasma membrane	CC	91	5.200871e-06
25.	GO:0005576	extracellular region	CC	73	1.857881e-05
26.	GO:0070013	intracellular organelle lumen	CC	53	9.636450e-04
27.	GO:0043233	organelle lumen	CC	53	9.636450e-04
28.	GO:0031974	membrane-enclosed lumen	CC	53	9.636450e-04
29.	GO:0005856	cytoskeleton	CC	51	2.194427e-03
30.	GO:0031981	nuclear lumen	CC	48	9.040712e-04

* *BP* Biological process, *MF* Molecular function, *CC* Cellular function

Among them, the most biologically relevant and statistically significant term was the cell cycle (GO:0007049), which is highly enriched and reflects a core disruption in cellular proliferation, likely due to stress-induced growth arrest or DNA repair (Fig. 5a). Similarly, DNA metabolic process (GO:0006259) and DNA recombination (GO:0006310) terms indicate increased genomic maintenance activity, suggesting that heat and hypoxia trigger DNA damage responses and repair pathways essential for cell survival. The enrichment of transmembrane transport (GO:0055085) points to altered membrane dynamics, potentially related to the ionic and nutrient balance critical during oxygen limitation. In terms of molecular function (Fig. 5b), binding (GO:0005488) and catalytic activity (GO:0003824) are associated with an increase in enzymes and molecular interactions, which is consistent with metabolic adjustments under stress. The term “membrane” (GO:0016020) from the cellular component category (Fig. 5c) emphasized structural and signaling modifications likely associated with cell environment interactions during hypoxia and thermal stress.

### KEGG pathway analysis

**Fig. 6:**
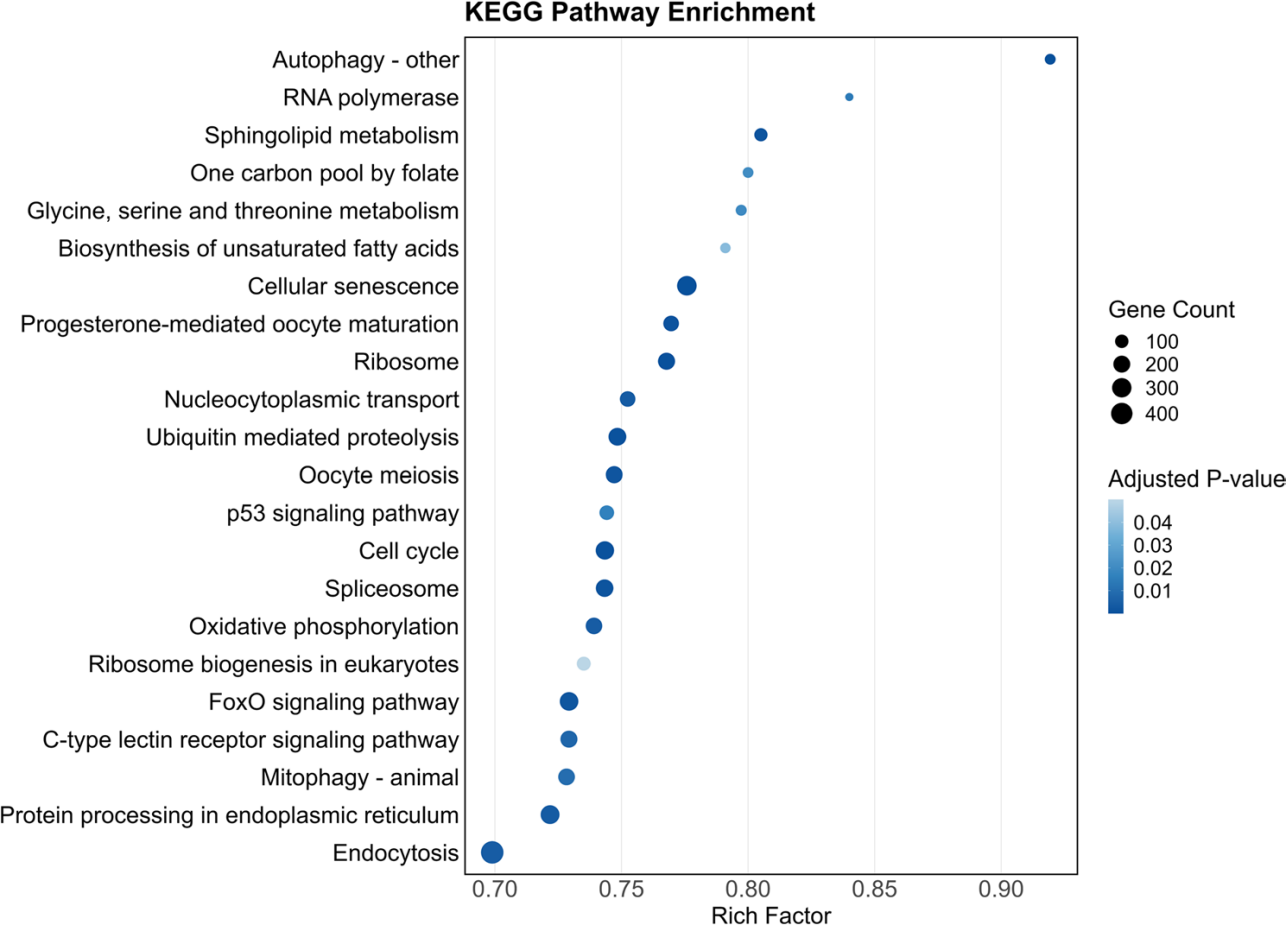
KEGG pathway enrichment analysis of DEGs under heat and hypoxia stress in rainbow trout (*O. mykiss*). The bubble plot shows the most significantly enriched KEGG pathways. The y-axis lists the enriched pathways, and the x-axis represents the rich factor (the ratio of differentially expressed genes to total genes in a pathway). The bubble dot size corresponds to the number of DEGs involved in each pathway, and the color intensity represents the adjusted p-value, with darker shades indicating greater statistical significance

Integrated analysis of heat and hypoxia stress responses in rainbow trout revealed 22 significantly enriched KEGG pathways (Fig. 6; Table 5), highlighting the coordinated cellular mechanism underlying the adaptation to combined environmental stressors. Cellular senescence (Rich factor = 0.776, p.adjust = 1.7E-07), p53 signaling (0.744, 0.01643), and the cell cycle pathway (0.743, 0.00051) collectively indicated the activation of cell cycle arrest and damage control mechanisms. FoxO signaling (0.729, 0.00167) further supported a shift toward stress resistance and survival pathways. Additionally, the increase in oxidative phosphorylation (0.739, 0.00401) suggested the involvement of mitochondria in energy adaptation under hypoxia, whereas protein processing in the endoplasmic reticulum (0.722, 0.00347) reflects the activation of the unfolded protein response to maintain proteostasis under thermal stress. A detailed list of significantly enriched KEGG pathways identified from the meta-analysis is provided in supplementary table S6.

**Table 5:** KEGG pathway enrichment analysis of DEGs. The table lists significantly top enriched biological pathways based on the KEGG database

ID	Description	Gene Ratio	Rich Factor	*p*.adjust	Count
omy04136	Autophagy - other	57/8897	0.91935	2.7E-05	57
omy03020	RNA polymerase	42/8897	0.84	0.01423	42
omy00600	Sphingolipid metabolism	95/8897	0.80508	0.00112	95
omy00670	One carbon pool by folate	56/8897	0.8	0.02092	56
omy00260	Glycine, serine and threonine metabolism	59/8897	0.7973	0.01971	59
omy01040	Biosynthesis of unsaturated fatty acids	53/8897	0.79104	0.03884	53
omy04218	Cellular senescence	308/8897	0.77582	1.7E-07	308
omy04914	Progesterone-mediated oocyte maturation	157/8897	0.76961	0.00082	157
omy03010	Ribosome	205/8897	0.76779	0.00014	205
omy03013	Nucleocytoplasmic transport	155/8897	0.75243	0.00347	155
omy04120	Ubiquitin mediated proteolysis	232/8897	0.74839	0.00051	232
omy04114	Oocyte meiosis	195/8897	0.74713	0.00158	195
omy04115	p53 signaling pathway	128/8897	0.74419	0.01643	128
omy04110	Cell cycle	255/8897	0.74344	0.00051	255
omy03040	Spliceosome	223/8897	0.74333	0.00112	223
omy00190	Oxidative phosphorylation	187/8897	0.73913	0.00401	187
omy03008	Ribosome biogenesis in eukaryotes	111/8897	0.7351	0.0495	111
omy04068	FoxO signaling pathway	264/8897	0.72928	0.00167	264
omy04625	C-type lectin receptor signaling pathway	202/8897	0.72924	0.00716	202
omy04137	Mitophagy - animal	193/8897	0.7283	0.0098	193
omy04141	Protein processing in endoplasmic reticulum	275/8897	0.72178	0.00347	275
omy04144	Endocytosis	462/8897	0.69894	0.00401	462

The GO enrichment of DNA metabolic process (GO:0006259, adj. *p* = 5.5 × 10⁻⁴) and cell cycle (GO:0007049, adj. *p* = 7.6 × 10⁻¹³) closely aligns with KEGG pathways such as Cell cycle (omy04110, adj. *p* = 5.1 × 10⁻⁴), p53 signaling (omy04115), and FoxO signaling (omy04068), indicating coordinated regulation of genome maintenance and cell division. Similarly, GO terms for nucleotide binding and membrane organization correspond to KEGG pathways for RNA polymerase (omy03020), spliceosome (omy03040), oxidative phosphorylation (omy00190), and endocytosis (omy04144), highlighting the link between transcription, protein synthesis, energy production, and intracellular transport during stress adaptation.

**Fig. 7:**
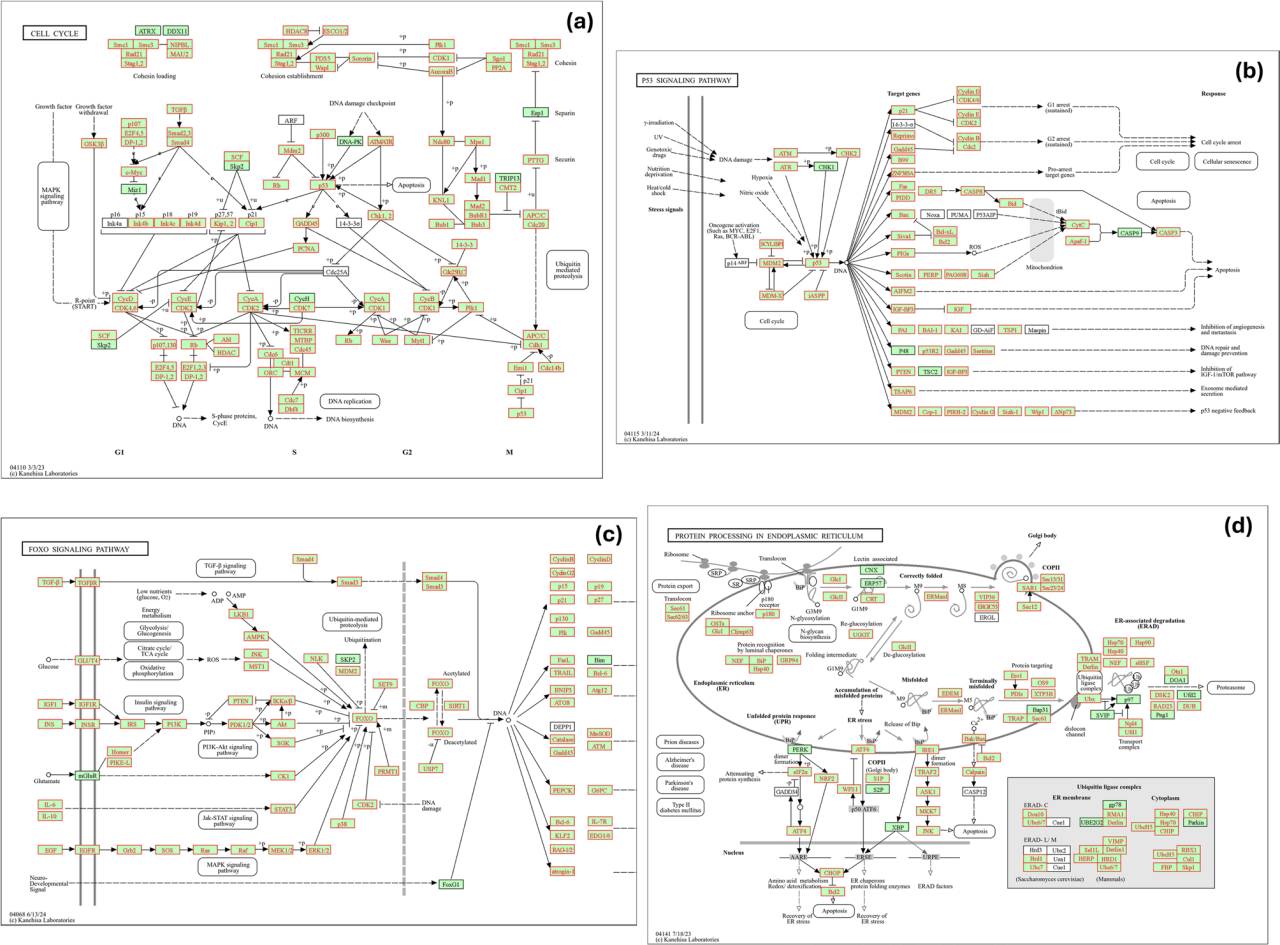
KEGG pathway maps of the four interconnected enriched pathways in response to heat and hypoxia stress in rainbow trout. Differentially expressed genes (DEGs), highlighted in red boxes, were identified through the meta-analysis and mapped onto four major signaling pathways **A** Cell cycle, **B** p53 signaling pathway, **C** FoxO signaling pathway, and **D** Protein processing in endoplasmic reticulum

Furthermore, to gain mechanistic insight into the most affected biological processes, Fig. 7 depicts four interconnected well-established signaling pathways: (a) the cell cycle, (b) the p53 signaling pathway, (c) the FoxO signaling pathway and (d) protein processing in endoplasmic reticulum. In the cell cycle (Fig. 7a), stress activates checkpoints at G1/S and G2/M phases by regulating key genes like *cdkn1a* (*p21*) and cyclins, delaying division to preserve genomic integrity. The p53 pathway (Fig. 7b) triggers DNA damage responses through effectors such as *p21* and *gadd45*, causing cell cycle arrest or apoptosis. Meanwhile, FOXO transcription factors (Fig. 7c) induce autophagy genes like *bnip3* and *atg12* under nutrient and oxidative stress, controlled by kinases *AMPK*, *JNK*, and *Akt*. In parallel, the ER stress and unfolded protein response (UPR) pathways, including the ER-associated degradation (ERAD) system, are activated (Fig. 7d), with chaperones such as BiP (*Hsp70*), *Hsp40*, and *Hsp90* upregulated to refold or degrade misfolded proteins.

## Discussion

The study revealed several key molecular chaperones, including *hspa1b*, *hspa4l*, *hspb3*, *serpinh1b*, *dnajb13*, and *hsf4*, were consistently upregulated across datasets representing thermal and hypoxic challenges in *O. mykiss*. These genes belong to the *hsp70*, *hsp40*, *small hsp*, *serpin*, and *hsf* families, indicating a conserved proteostasis mechanism [48]. Specifically, both *hspa1ba* and *hspa4l*, which are part of the *Hsp70/110* gene family, were found to be upregulated at the transcript and protein levels in liver and head kidney tissues during heat stress [49, 50]. The consistent upregulation of *serpinh1b* in the liver of rainbow trout suggests its protective role in maintaining extracellular matrix stability and facilitating tissue repair under thermal and pathogenic stress [51]. Additionally, functional studies in zebrafish demonstrate that *hsf4* is crucial for tissue-specific proteostasis rather than for early embryonic lens formation [52]. The persistent upregulation of these chaperones underscores their central role in managing protein misfolding and reducing cellular damage in response to multifactorial environmental stress in *O. mykiss*, with similar patterns observed in zebrafish [53], mrigal [54], atlantic cod [55], yellow croaker [56], and goldfish [57]. The study also revealed hypoxia-responsive genes such as *egln3*, *bnip3*, *hk2*, *pfkfb3*, and *glut1a* reflect the activation of HIF-mediated metabolic pathways under stress [58]. In *O. mykiss*, *egln3*, an oxygen-sensing prolyl hydroxylase, is broadly expressed under normal conditions but downregulated by hypoxic condition in most tissues [59], where *glut1a*, a key glucose transporter, is upregulated to enhance cell viability [60]. In contrast, *saa* and *nek2* were found as most down regulated genes, reflecting suppressed acute-phase immunity [61] and cell cycle activity [62]. The suppression of the acute-phase gene *saa* indicates an energy trade-off during prolonged stress, prioritizing essential metabolic and protective functions over immune defense.

The meta-analysis identified that the genes *cdkn1b*, *znf395a*, and *klf9* were differentially expressed across all four datasets. *Cdk* inhibitors, such as *cdkn1b* (*p27*), enforce cell cycle arrest at the G1/S checkpoint, allowing time for DNA repair [63, 64]. Its upregulation in *Tor putitora* during heat stress highlights its role in thermotolerance and prevention of apoptosis [65]. In zebrafish, *cdkn1c* is regulated by Hedgehog signaling, which coordinates stress-induced differentiation with Myod during development [66]. *Znf395a* functions as a hypoxia-inducible transcription factor with zinc finger domains that regulate stress-responsive gene expression [67]. Its expression under cyclic and chronic hypoxia contributes to inflammation [68], tumor survival [69], and interacts with the HIF-1α [70] and NF-κB signaling pathways [71, 72], reinforcing its role in the stress-adaptive transcriptional network. Lastly, *klf9*, a conserved stress-responsive transcription factor, modulates development, apoptosis, and glucocorticoid signaling in response to oxidative and environmental stimuli [73]. Among these genes, knocking out *cdkn1ba* could clarify its specific role in cellular homeostasis and stress adaptation.

Pathway enrichment analysis reveals interconnected regulatory hubs, including p53 signaling, the cell cycle, and cellular senescence, which coordinate cellular stress responses by directing cell fate toward repair, apoptosis, or senescence. The enriched p53 signaling pathway is a central regulator of cell cycle arrest and apoptosis during DNA damage [74], which aligns with in vitro findings identifying *p53* and γH2AX as sensitive biomarkers of genotoxic stress in *O. mykiss* [75]. This role of p53 is further supported across species, being activated by miR-34a under hypoxia in genetically improved tilapia [76], linked to cold stress responses in large the yellow croaker [77], and associated with disease resistance via cd82a-mediated regulation [78], highlighting its conserved importance in fish stress adaptation and immune defense. Following the activation of these core mechanisms, the enrichment of both the cell cycle and cellular senescence pathways indicates that stress first slows cell division through checkpoint arrest [79], and if damage continues, cells shift into a senescent state [80] to prevent further genomic instability [81], which eliminates damaged cells during development or environmental stress [82, 83]. In rainbow trout hepatocytes, senescence-like features have been observed under chronic chemical exposure [84]. The results of zebrafish further support its role in regeneration and adaptation to oxidative stress [85, 86]. Senescence also intersects with membrane dynamics and phospholipid remodeling, which are essential under temperature stress [87]. Moreover, the significant presence of protein processing in the endoplasmic reticulum and endocytosis pathways highlights the mechanisms that maintain proteostasis during heat-induced protein unfolding. Under thermal stress, rainbow trout liver cells activate ER-associated degradation (ERAD) and upregulate the expression of chaperones, ERAD components, and UPR genes to eliminate misfolded proteins and maintain cellular homeostasis [88]. Endocytosis, particularly fluid-phase endocytosis, is highly temperature sensitive but compensates in rainbow trout hepatocytes by modifying membrane lipids and vesicle trafficking proteins [89]. This ensures continuous nutrient and signal uptake across acclimation states, underscoring the importance of membrane remodeling in maintaining cellular function under thermal stress [90].

Another enriched FoxO signaling pathway involved in regulating autophagy, apoptosis, cell cycle arrest, and metabolic adaptation [91, 92]. FOXO transcription factors are primarily activated under stress rather than normal physiological conditions, enabling cells to maintain homeostasis during environmental and metabolic challenges [93]. In mammals, the link between FoxO signaling and autophagy is multifaceted; for example, *FOXO3* can enhance *FOXO1*-driven autophagy via PIK3CA–AKT1 signaling [94], while acetylated *FOXO1* directly interacts with *atg7* to promote autophagic flux independent of transcription during hypoxia [95]. In fish, hypoxia-induced oxidative stress promotes autophagy in grass carp hepatocytes by inhibiting Akt phosphorylation and activating the *FoxO1* pathway [96]. In skeletal muscle, *FOXO3* initiates both lysosomal autophagy and proteasomal degradation, with *bnip3* as a key effector independent of the proteasome [97, 98]. Similarly, *FOXO1/3* reduce cardiomyocyte size under glucose starvation or ischemia by inducing autophagy through sirt*1* and *rab7a* signaling [99]. These mechanisms highlight the conserved role of *FOXO* in regulating autophagy during metabolic stress [100, 101]. Moreover, In ectothermic species, FoxO signaling also integrates metabolic and apoptotic responses under temperature stress: cold exposure in zebrafish and tilapia reveals species-specific regulation of apoptosis via a FoxO-centered network [102], while in Tsinling lenok trout, thermal stress triggers FoxO-mediated mitochondrial apoptosis and impairs liver function [103]. Collectively, these findings support FoxO’s role as a central hub linking environmental stress to autophagy and apoptosis across vertebrates. Overall, the integrated analysis suggests that rainbow trout respond to both heat and hypoxia stress through a conserved network of cellular defense mechanisms converging on stress-sensing and metabolic regulatory pathways.

A limitation of this meta-analysis is that the included datasets represent a relatively narrow size range of rainbow trout (200–400 g) and are limited to liver and muscle tissues. This may not capture size or developmental stage specific transcriptional responses that could occur in smaller juveniles or larger adults and may also overlook tissue-specific stress adaptation mechanisms in other organs such as gills or brain, which are critical for thermal and hypoxia tolerance.

## Conclusion

Rainbow trout responds and adapts itself to heat and hypoxia stress via a conserved network of cellular defense mechanisms, which is reflected in the coordinated regulation of DEGs. These genes, involved in the cell cycle, protein processing in the endoplasmic reticulum, FoxO signaling, and oxidative stress responses, form a shared molecular program that maintains proteostasis and cellular homeostasis under environmental stress. Overall, these findings deepen our understanding of rainbow trout’s molecular stress responses and provide promising targets for future functional studies to enhance aquaculture resilience.

## Supplementary Information

Supplementary material 1.

Supplementary material 2.

Supplementary material 3.

Supplementary material 4.

Supplementary material 5.

Supplementary material 6.

## Data Availability

The data supporting the findings of this study are included within the article and its supplementary information files. Gene expression datasets related to heat and hypoxic stress (PRJNA1092638, PRJNA559610, PRJNA1064938, and PRJNA1000995) were retrieved from the NCBI Gene Expression Omnibus (GEO) database (https://www.ncbi.nlm.nih.gov/geo/). All scripts used in this study are publicly available on GitHub: https://github.com/BariSayed/Omyk_Stress_MetaRNASeq and https://github.com/bigbiolab/Omyk_Transcriptomic_Meta.
